# Toxoplasmosis complications and novel therapeutic synergism combination of diclazuril plus atovaquone

**DOI:** 10.3389/fmicb.2014.00484

**Published:** 2014-09-15

**Authors:** Helieh S. Oz

**Affiliations:** Department of Internal Medicine, University of Kentucky Medical CenterLexington, KY, USA

**Keywords:** toxoplasmosis, combination, diclazuril, atovaquone, synergism, obesity, *Toxoplasma*, gastroenteritis

## Abstract

Toxoplasmosis is a major cause of foodborne disease, congenital complication, and morbidity. There is an urgent need for safe and effective therapies to encounter congenital and persisting toxoplasmosis. The hypothesis was: combination diclazuril plus atovaquone to exert a novel therapeutic synergy to prevent toxoplasmosis syndromes.

**Methods:** Pregnant dams were treated with diclazuril and atovaquone monotherapy or combination therapy and infected i.p with *Toxoplasma* tachyzoites.

**Results:** Infected dams developed severe toxoplasmosis associated syndrome with increases in the abdominal adiposity surrounding uteri, gansterointestinal and other internal organs and excessive weight gain. Numerous organisms along with infiltration of inflammatory cells were detected scattered into adipose tissues. Combination therapy (*p* < 0.01) and to a lesser extent diclazuril (*p* < 0.05) protected dams from inflammatory fat and excess weight gains. This was consistent with pancreatitis development in infected dams (versus normal *p* < 0.05) with infiltration of inflammatory cells, degeneration and necrosis of pancreatic cells followed by the degeneration and loss of islets. Combination and monotherapy protected dams from these inflammatory and pathological aspects of pancreatitis. Infected dams exhibited severe colitis, and colonic tissues significantly shortened in length. Brush border epithelial cells were replaced with infiltration of lymphocytes, granulocytes, and microabscess formations into cryptic microstructures. Combination therapy synergistically preserved colonic structure and normalized pathological damages (*p* < 0.001) and to a lesser degree diclazuril monotherapy protected dams from colitis (*p* < 0.05) and gastrointestinal toxoplasmosis. Other complications included severe splenitis (*p* < 0.001) and hepatitis (*p* < 0.001) which were normalized with combination therapy.

**Conclusion:** Combination diclazuril plus atovaquone was safe and with a novel therapeutic synergism protected dams and fetuses from toxoplasmosis.

## INTRODUCTION

Toxoplasmosis is a major cause of foodborne disease, hospitalization, and congenital complications related morbidity and mortality (Mead et al., 1999; Scallan et al., 2011; Hoffmann et al., 2012). *Toxoplasma* is categorized as class B human pathogen by the CDC and NIH. Toxoplasmosis, a cosmopolitan syndrome, is considered as “forgotten disease of vulnerable and poverty” which infects the many in rural (Hotez, 2008) as well as the urban areas. Congenital toxoplasmosis is due to transmission of *Toxoplasma* organisms from infected mom to the fetus and typically associated with pregnancy immunosuppression. Congenital toxoplasmosis causes severe complications in fetal and neonate to compromise a lifelong adverse consequences (Remington et al., 2004; Olariu et al., 2011; Kieffer and Wallon, 2013). *Toxoplasma* organisms are transmitted through consumption of contaminated meat, milk, dairy product with cysts forms. However, the main source of *Toxoplasma* infection is considered as vegetables, fruits, and water contaminated with oocysts from cat feces in the field, while over 93 million cats are kept as pets in the USA. These households may include immunocompetent as well as immunosuppressed, obese and/or diabetic and pregnant individuals, and at risk of developing toxoplasmosis (Esch and Petersen, 2013).

Toxoplasmosis, a global disease, and in excess of billion people are expected to have *Toxoplasma* infection. *Toxoplasma* is associated with anorexia or obesity as organisms alter and reside in inflamed adipose tissues (Carter, 2013). Excessive weight gain is reported in infected pregnant women compared with uninfected individuals (Kankova et al., 2010; Flegr, 2013), as well as in a feto-maternal toxoplasmosis model (Oz and Tobin, 2012; Oz, 2014). *Toxoplasma* infected animals had increased weight gain and atrophy of myenteric neurons of the jejunum (Hermes-Uliana et al., 2011). Obesity has become a cosmopolitan syndrome and poorly understood pathogenesis with a potential link to toxoplasmosis. Other toxoplasmosis complications are gastroenteritis, pancreatitis, diabetes, retinochoroiditis, and encephalitis.

Current available therapy for congenital toxoplasmosis is spiramycin associated with pyrimethamine plus sulfadoxine combined therapy, to protect fetus from *Toxoplasma* organism transmission in actively infected moms. However, this approach is not always effective and the treatment has fetotoxic side effects (Habib, 2008; Berrebi et al., 2010; Cortina-Borja et al., 2010; Julliac et al., 2010). Pyrimethamine while used is a pregnancy classified C drug, which may cause bone marrow suppression in the mom and the newborn. In a clinical trial in France, 24% of sera positive women treated with spiramycin and pyrimethamine plus sulfadoxine combination delivered *Toxoplasma* infected infants (Bessieres et al., 2009). Spiramycin monotherapy can be effective only when administered during early stage of pregnancy and is principally a preventive measure (Julliac et al., 2010). More than half of patients treated with spiramycin retained *Toxoplasma* DNA in their blood and remained infected (Habib, 2008). Fifty-five percent of patients treated with combination of sulfadiazine + pyrimethamine plus folinic acid therapy have adverse effects (Capobiango et al., 2014). Meanwhile, the efficacy of azithromycin, clarithromycin, atovaquone, dapsone, and cotrimoxazole (trimethoprim-sulfamethoxazole), has not been clinically proven (Petersen and Schmidt, 2003). Considering the importance of complications and the worldwide epidemic, there is an urgent need for effective and nontoxic therapeutic modalities for congenital or persisting chronic toxoplasmosis.

Diclazuril and its related benzeneacetonitriles have been used in treatment and prevention of livestock and poultry coccidiosis (Assis et al., 2010) and *S. neurona* in EPM. Diclazuril is a safe and effective compound at therapeutic dose levels (Assis et al., 2010; Oz and Tobin, 2014). Diclazuril targets chloroplast derived chlorophyll a-D1 complex present in *Toxoplasma* and other Apicomplexans and not exists in mammalians cells (Hackstein et al., 1995).

Atovaquone is a FDA approved toxoplasmosis treatment but not in feto-maternal toxoplasmosis (Cortina-Borja et al., 2010; Oz and Tobin, 2012; Oz, 2014). Atovaquone is a safe and effective drug against plasmodial infections (Hudson et al., 1991), *Babesia microti,* causative of human babesiosis (Hughes and Oz, 1995; Oz and Westlund, 2012) and other opportunistic disease, *Pneumocystis* pneumonia (Oz et al., 1999).

Recently, the efficacy of diclazuril and atovaquone monotherapy were reported against inflammatory and infectious aspects of mild to moderate feto-maternal toxoplasmosis (Oz and Tobin, 2012, 2014; Oz, 2014). Therapeutic diclazuril plus atovaquone combination have not been previously reported against colitis, pancreatitis and some other inflammatory complications in toxoplasmosis. This investigation explores the efficacy of combination therapy with diclazuril plus atovaquone to exert a novel therapeutic synergism to protect against toxoplasmosis.

## MATERIALS AND METHODS

### ETHICS

This research was conducted according to the guidelines and approved by the IBC and the Care and Use of Laboratory Animal Care (IACUC) at University of Kentucky Medical Center.

### *Toxoplasma gondii* PROPAGATION

*Toxoplasma* Type II isolates including ME-49 strain are reported predominant in human congenital Toxoplasmosis (Ajzenberg et al., 2002). For this investigation, *Toxoplasma* organisms from PTG strain (ME-49, ATCC50841) were originally cloned and propagated by Dr. Daniel Howe of the Maxwell H. Gluck Equine Research Center at the University of Kentucky (Howe et al., 1997; Oz and Tobin, 2012). Briefly, Tachyzoites were cultured by serial passage in bovine turbinate cells and maintained in MEMRS (HyClone Labs, Inc.) supplemented with 4% fetal clone III (HyClone, Labs, Inc.), Penicillin/streptomycin/fungizone (BioWhittaker, Inc.), and nonessential amino acids solution (HyClone, Labs, Inc.). Upon disruption of the host cell monolayer, extracellular tachyzoites were harvested and purified from host cell debris by filtration through 3.0 μm membranes. Tachyzoites were enumerated in a hemocytometer and suspended in PBS to the appropriate concentrations for inoculation. All inoculations were administered i.p. in 100 μL volume into dams within 1 h of harvesting to ensure viability.

### CONGENITAL TOXOPLASMOSIS MODEL

Day 1 programmed pregnant (9 weeks old) CD1 mice were purchased from Charles River Lab Inc., Wilmington, MA, USA). Dams were housed individually in microisolator cages in a pathogen free environment and maintained at 22^∘^C with a 12: 12 h light: dark cycle at the Maxwell H. Gluck Equine Research Center Laboratory Animal Facility. Animals were fed irradiated rodent chow and sterilized drinking water *ad libitum*. After 5 days acclimation, dams were weighed and ear punched for appropriate identification. They were assigned into 6–8 animals per group and injected i.p. with 100 μL PBS containing 0 or 600 tachyzoites with 0.5 mL insulin syringes. Control dams received 100 μL injection with PBS alone (Oz and Tobin, 2012). Animals were monitored daily three times for distress, pain, physical appearance, and vaginal discharge to detect abortion or early delivery (Oz and Tobin, 2012, 2014). The experiment was terminated on gestation day 16 before possible early or premature birth to study the fetal and maternal aspects of the disease.

### SPECIMENS COLLECTION

Animals were euthanatized using CO_2_ inhalation. Immediately their chests were opened and blood from heart collected in microtainers (BD Biosource, Rockville, MD, USA) for hematocrit evaluation. Sera were separated and stored at frozen –80^∘^C. The splenic weight and length were recorded. Heart, liver, and uterus were excised and weighed. Colonic contents were removed and colonic length and weigh data measured and flash frozen in liquid nitrogen and stored at –80^∘^C for future studies. Live fetuses were removed from uteri, counted, and weighed and their lengths measured using a digital caliper. All aspects of the investigation were performed according to the guidelines by Institutional Biosafety Committee (IBC) and IACUC at University of Kentucky Medical Center.

### DICLAZURIL AND ATOVAQUONE THERAPIES

To study safety and efficacy of diclazuril plus atovaquone against toxoplasmosis, dams were divided into groups of 18–24. Dams received regimens, diclazuril monotherapy, atovaquone monotherapy, diclazuril plus atovaquone combination therapy, or sham incorporated into daily diet (Oz et al., 2007; Oz and Tobin, 2012, 2014). The control group received sham treatment (inert talcum powder). Treatments were initiated on Day 5 of pregnancy and continued until day 16. On day 8 of pregnancy dams on treatments or sham control arms were further divided into three subgroups of 6–8 animals and were injected each with PBS alone, or PBS containing 600 tachyzoites and treatments were continued until dams were euthanatized. Pregnant animals voluntarily consumed their diets with no significant changes in their appearance, food consumption, or weight loss/gain.

### PATHOLOGICAL ASSESSMENTS

#### Hematoxylin eosin staining

A portion of examined tissues from each dam was placed into cassettes and fixed with 10% neutral PBS formalin. The specimens were dehydrated and embedded in paraffin, and tissue sections of 5 μm were stained by H&E for histopathological evaluation.

#### Giemsa staining

Giemsa is a delicate polychromatic stain that reveals the fine nuclear detail of *Toxoplasma* organisms (Oz and Tobin, 2012). Giemsa stain contains methylene blue azure basic (MBAB) dyes combined with eosin acidic dyes. The deparaffinized slide sections were stained with the polychromatic Giemsa (40 drops/50 mL distilled water) to stain nuclei of the *Toxoplasma* organisms and to permit differentiation among the cells. Then, the slides were depreciated in 1% glacial acetic acid, dehydrated in alcohol and xylene series, and mounted in synthetic resin on slides.

#### Immunohistochemical staining (IHC)

Anti-*Toxoplasma* antibody and IHC procedure were kindly provided by Dr. David S. Lindsay at University of West Virginal. Briefly, paraffin-embedded sections were cut, deparaffinized with xylene, rehydrated in alcohol baths, washed in PBS with 0.1% BSA, quenched endogenous peroxidase activity by incubating in 3% hydrogen peroxide in methanol for 30 min, and then blocked with rabbit serum (Dako number 1699), 30 min. The sections were incubated with polyclonal Rh anti-*Toxoplasma* antibody, diluted 1: 500 for 90 min, and developed with DAB-chromogen (Dako, Carpinteria, CA, USA) for about 5 min until signal developed and subsequently counterstained with hematoxylin then ammonia treated dehydrate stepwise through alcohol, clear with xylene (Oz and Tobin, 2012, 2014).

### COLONIC TISSUES PREPARATION AND EVALUATION

Colonic tissues were flushed with PBS (pH 7.2) and a portion from proximal and distal colonic tissue was fixed in 10% neutral formalin for histological examinations. The remainder was flash-frozen in liquid nitrogen and stored at –80^∘^C. The formalin fixed sections were processed and stained with H&E and slides evaluated by Ziess light microscopy. The severity of colitis as assessed with a histological semiquantitative grading score and performed in a blinded fashion. The scores were based on histopathological features with a numeric value (0: normal to 4: severe) assigned according to the tissue involvement that corresponded to either of the following criteria (Oz et al., 2007, 2010, 2013).

(Grade 0)—no detectable lesions, no inflammatory cells, and normal mucosal appearance.
(Grade 1)—focal inflammatory infiltrate in the mucosa.
(Grade 2)—mild multifocal inflammation with moderate expansion of the mucosa.
(Grade 3)—moderate multifocal inflammation with moderate expansion of the mucosa.
(Grade 4)—severe diffuse inflammation with crypt epithelium disruption and ulceration.

### ADIPOSITY TISSUE PREPARATION AND STAINING

Portions of the abdominal adipose tissue from each dam were removed, placed in a cassette and fixed in the 10% buffered formalin and processed for histophathological slides staining with Giemsa, IHC, and H&E to study the structure and possible organisms.

### HEPATIC TISSUE PREPARATION AND STAINING

A portion of the right lobe from liver tissues of each dam was placed in cassette and fixed with 10% neutral PBS formalin. The specimens were dehydrated and embedded in paraffin, and tissue sections of 5 μm were stained by H&E. Each slide was evaluated under Ziess light microscopy. Hepatic lesions were graded on a scale of 0–4 + based on degeneration, inflammation, and necrosis (Oz et al., 2006, 2011) as follows.

(Grade 0)—no detectable lesions, no degeneration, infiltration of inflammatory cells, and normal tissue appearance.
(Grade 1)—focal infiltration of inflammatory cells in the tissue and hepatocytes degeneration.
(Grade 2)—mild multifocal infiltration of inflammatory cells, and hepatocytes degeneration.
(Grade 3)—moderate multifocal infiltration of inflammatory cells and hepatocytes degeneration.
(Grade 4)—severe diffuse infiltration of inflammatory cells and necrosis.

### PAIN RELATED BEHAVIORAL TEST

Assessment of Pain Related Mechanical Allodynia by Testing Abdominal Withdrawal Threshold. Abdominal withdrawal responses to mechanical stimuli were quantified with von Frey monofilaments (Semmes-Weinstein Anesthesiometer Kit) according to our previous publications with some modification (Oz and Tobin, 2012, 2014). Dams were placed into plastic enclosures on the custom-made screen meshed platform. The monofilament range used for this study included five different intensities corresponding to (hair diameter) gram force [(4.08) 1.0 g; (3.61) 0.4 g; (3.22) 0.166 g; (2.83) 0.07; (2.36) 0.02 g forces]. Testing for mechanical stimulation was performed on the first and the last days of treatment. A single trial consisted of five applications of the each filament used once every 6 s to allow dam to cease any response and return to an inactive position. Mean values of the percentage of responses of the abdominal withdrawal to each filament (mean withdrawal/5 × 100) were used as % scores for this study. This behavioral test reflected basal level for reflex score and any possible sensory changes observed in the treated mice. A total of four dams were tested per each group.

### STATISTICAL ANALYSIS

Results are expressed as mean ± SEM unless otherwise stated. Data were evaluated with ANOVA followed by appropriate *post hoc* test (Tukey compared all pairs) using GraphPad Instat version 3 for Windows (Graph-Pad Software, San Diego, CA, USA). Statistical significance was set at *p* < 0.05.

## RESULTS

In the preliminary trial, groups of naïve dams were treated with diclazuril monotherapy, atovaquone monotherapy, diclazuril plus atovaquone combination therapy, or inert talcum sham treatment. Dams consumed medicated diets with no detectable side effects such as changes in physical appearance, appetite, food consumption, and the rate of weight gain or fetotoxicity and abortion.

### TOXOPLASMOSIS AND INFLAMMATORY ADIPOSITY

**FIGURE 1:**
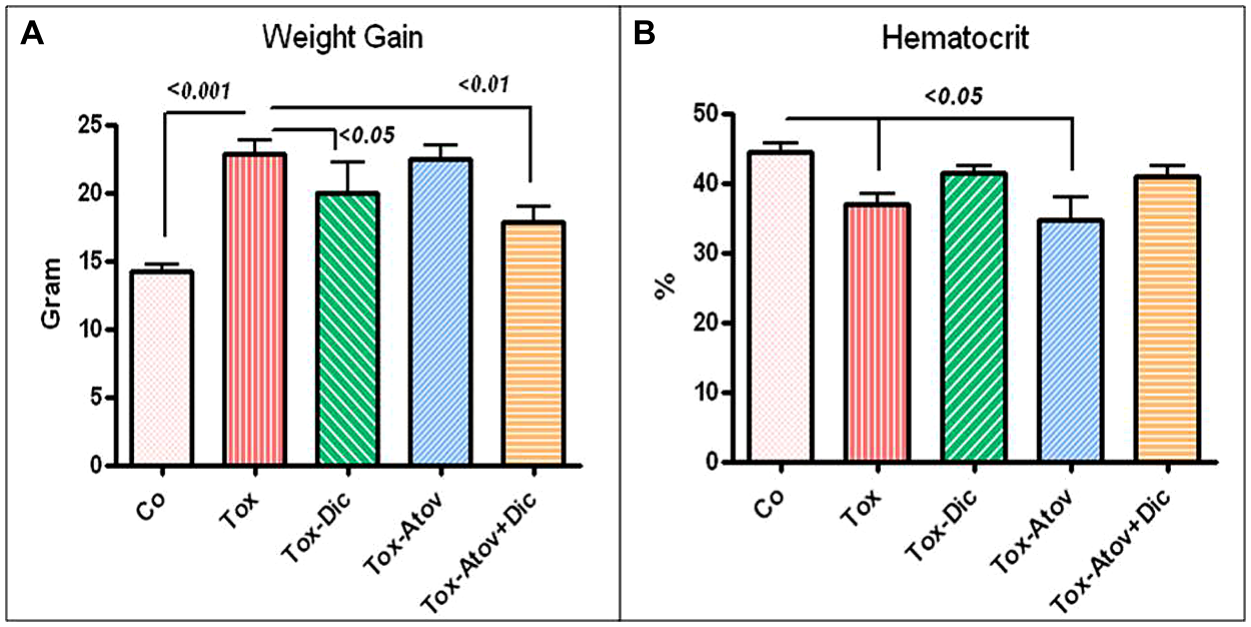
**(A)** *Toxoplasma* infection caused significant anemia in sham treated dams (Tox). Combination diclazuril plus atovaquone therapy (Dic + Atov) and diclazuril monotherapy (Dic) protected dams but atovaquone (Atov) monotherapy had no effect. **(B)** Body weight gain during pregnancy in normal sham controls (Control) compared with excess pathological weight due to accumulation of inflammatory fat in *Toxoplasma* infected (Tox) sham treated dams (*p* < 0.001). Combination therapy with diclazuril plus atovaquone synergistically protected dams (*p* < 0.01) and to a lesser extent diclazuril monotherapy (*p* < 0.05) prevented pathological fat accumulation and excess weight gain. Atovaquone monotherapy had no significant effect (*n* = 6–8/group).

**FIGURE 2:**
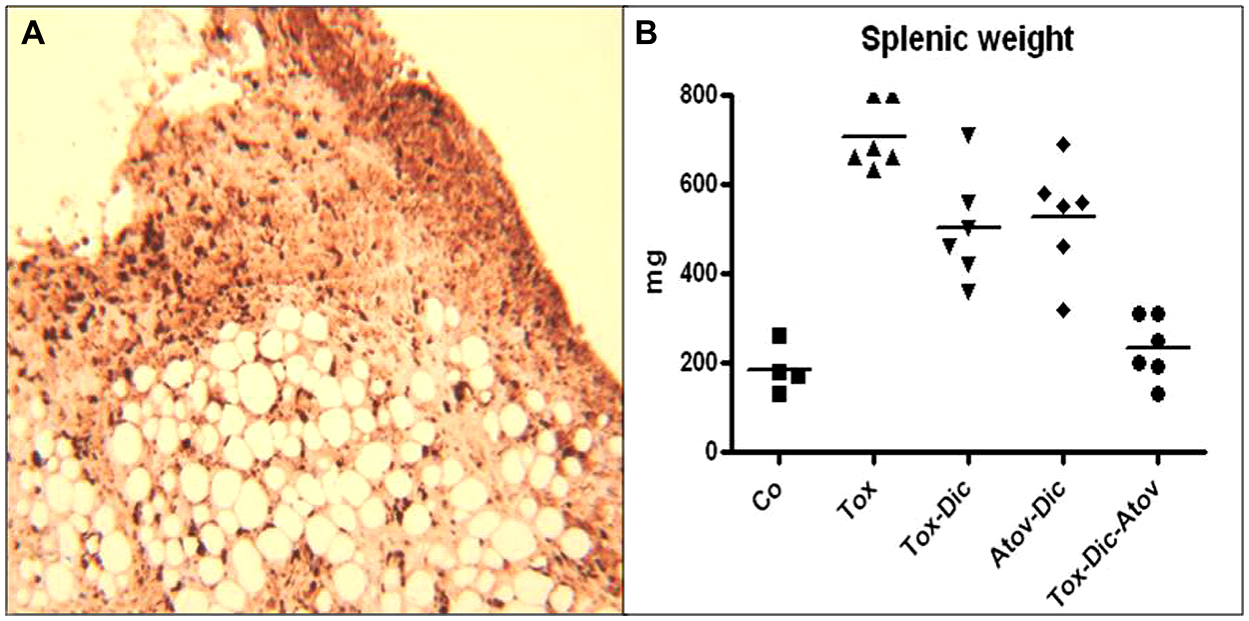
**(A)** Demonstrates inflammatory fat depot located in abdominal cavity and harboring numerous organisms. IHC stained *Toxoplasma* organisms (dark brown). (*n* = 6 group). **(B)** Spelenic weight distribution in *Toxoplasma* (Tox) infected (*p* < 0.001) compared to diclazuril (Dic) monotherapy (*p* < 0.01), atovaquone (Atov), synergistic effect of diclazuirl plus atovaquone (Dic + Atov) combination therapy (*p <* 0.001*)* and normal sham controls (Co). (*n* = 6/group).

For the next investigation, groups of dams were treated with (a) diclazuril monotherapy, (b) atovaquone monotherapy (c) diclazuril plus atovaquone combination therapy, or (d) received sham treatment. Then each group was further subdivided and injected with sham, or a dose of 600 tachyzoites. Infected dams developed *Toxoplasma* infection (600 tachyzoites) versus uninfected normal controls received sham (PBS) injection. The infected-sham treated dams showed a progressive severe toxoplasmosis complications including anemia, hydrothorax, and ascities (*p* < 0.05). Combination therapy with diclazuril plus atovaquone and diclazuril monotherapy protected dams from anemia, hydrothorax, and ascites (**Figure 1A**). Normal-sham injected and sham-treated controls (control) gained body weight during pregnancy compared with excessive pathological weight gain due to accumulation of inflammatory adiposity in *Toxoplasma-*infected (Tox) sham treated dams (*p* < 0.001). Combination therapy with diclazuril plus atovaquone synergistically protected dams (*p* < 0.01) and to a lesser extent diclazuril monotherapy (*p* < 0.05) prevented pathological accumulation of adipose tissues and excess weight gain. In contrast, atovaquone monotherapy had no significant effect on the weight gain and accumulated adiposity (**Figure 1B**). Massive inflammatory adipose depot was detected in the abdominal cavity surrounding uteri and gastrointestinal and kidneys. The adipose tissues were shown to harbor numerous inflammatory cells in H&E stainings as well as *Toxoplasma* organisms as confirmed with Giemsa and IHC stainings (**Figure 2A**). Organisms were not detectable in dams with combination therapy.

### *Toxoplasma* INDUCED SPLENITIS

Splenic tissues enlarged significantly and increased in weight and length in infected-sham treated dams. Enlarged splenic tissues from *Toxoplasma* infected dams showed significant infiltration of epithelioid cells and multinucleated giant cells with loss of germinal structure and caused a severe splenomegaly. *Toxoplasma* organisms were detected in IHC staining. Combination therapy diclazuril plus atovaquone synergistically prevented dams from severe splenitis and tissue damages (*p* < 0.001), **Figure 2B**, **Table 1**.

**Table 1:** Efficacy of diclazuril and atovaquone monotherapy or combination treatment on toxoplasmosis.

Tissues	Control	Tox	Tox + Dic	Tox + Atov	Tox + Dic + Atov
Fetal weight	700 ± 40	530 ± 14^c^	650 ± 25^b^	710 ± 25	720 ± 20
Splenic length (mg)	2.28 ± 013	3.22 ± 0.2^c^	2.8 ± 0.18	3 ± 0.1^a^	2.3 ± 0.13^b^
Pain score*(%)	20 ± 6	43 ± 3^b^	40 ± 4^b^	25 ± 2.9	25 ± 2.8

Tissues from normal sham treated and PBS containing no tachyzoites injected controls (*Control)*, infected-dams with *Toxoplasma* tachyzoites and treated with sham (Tox), compared with infected dams from diclazuril monotherapy (Tox + Dic), Atovaquone monotherapy (Tox + Atov), or combination diclazuril plus Atovaquone (Tox + Dic + Atov) therapy. Dams were monitored daily three times until day 16 of pregnancy before termination. Number 6–8/each group.

*Percent abdominal pain related behavioral response to von Frey stimuli with 0.166 GM force. Abdominal hypersensitivity significantly increased in infected dams (Tox). Combination therapy (Atov + Dic + Atov) and atovaquone monotherapy (Tox + Atov) similarly normalized pain induced behavioral modification in dams, but diclazuril monotherapy (Tox + Dic) had no effect. ^a^*p* < 0.05; ^b^*p* < 0.01; ^c^*p* < 0.001.

### *Toxoplasma* INDUCED COLITIS

**FIGURE 3:**
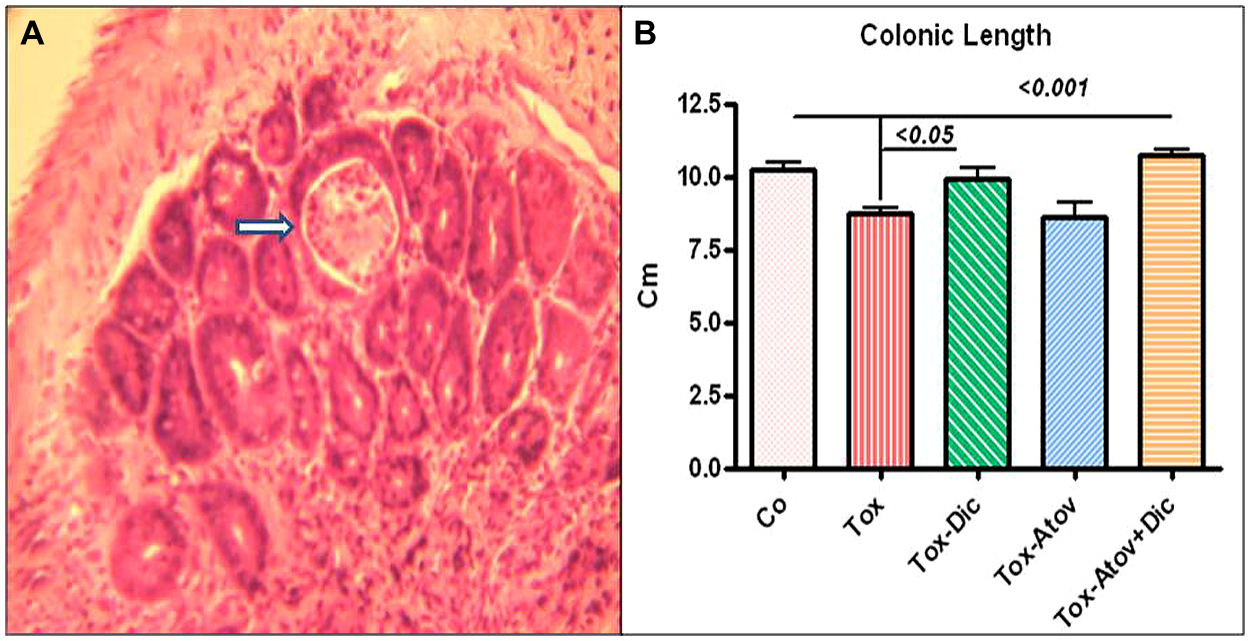
**(A)** Colonic section stained with H&E from *Toxoplasma* infected sham treated dam (Tox) developed severe colitis with destruction of brush border, and loss of colonic epithelial cells, microabscess formation (open arrow) and infiltration of inflammatory cells into mucosa (*n* = 6 /group). **(B)** Colonic length shortened due to infiltration of inflammatory cells, and microabscess formation in infected sham treated dams (*p* < 0.001). Combination therapy with diclazuril plus atovaquone (Dic + Atov) preserved colonic structure and to a lesser extent Diclazuril (Dic) monotherapy improved the colitis (*p* < 0.01). (*n* = 6–8/group).

Colonic tissues from infected-sham treated dams were significantly shortened in length (10.4 ±0.2 vs. infected 8.7 ±0.6 cm, *p* < 0.001) but decreased in weight (*p* < 0.01), presumably through the mechanism of sloughing off of the brush boarder due to infection (**Figure 3A**). Colonic pathology manifested with shortening of crypts with numerous microabscess formations in the cryptic structures and infiltration of inflammatory cells, including lymphocytes, with scattered neutrophils detected in the mucosal architecture. Combination therapy synergistically prevented pathologic changes (*p* < 0.001) and to a lesser extent diclazuril monotherapy (*p* < 0.05) preserved the colonic length and weight and the integrity of the microstructure against inflammatory response (**Figure 3B**). In contrast, atovaquone monotherapy had no significant protective effect on colonic inflammation and necrotic/atrophic responses to the infection.

### *Toxoplasma* INDUCED HEPATITIS

**FIGURE 4:**
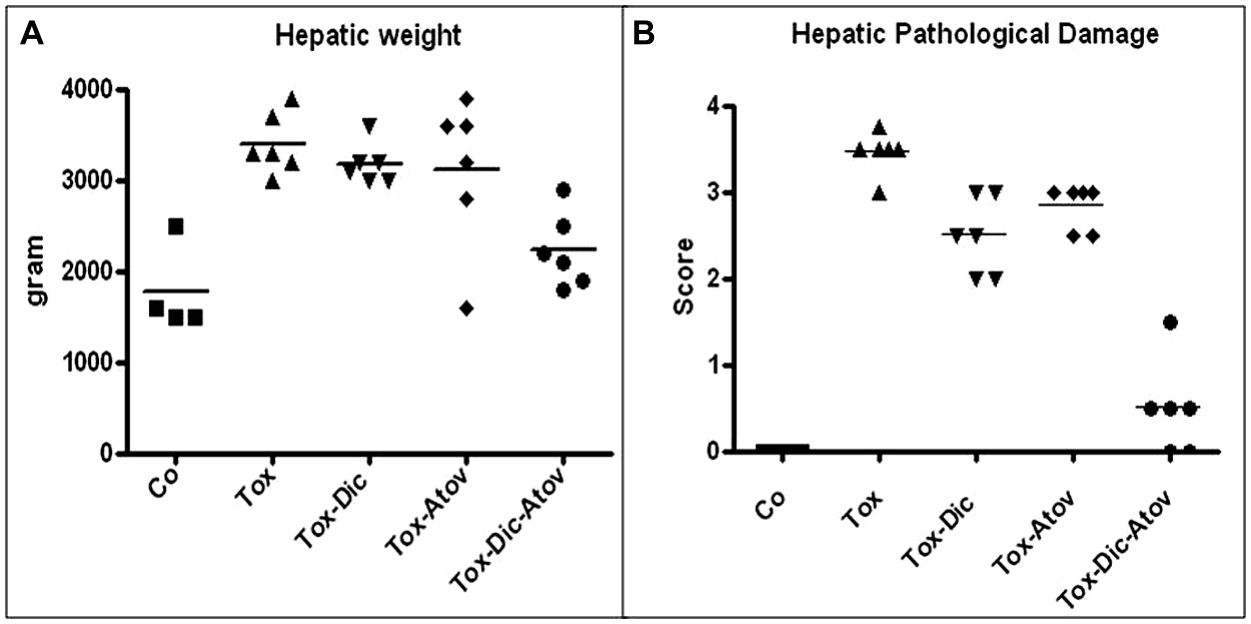
**(A)** Hepatic weight distribution in *Toxoplasma* (Tox) infected (*p* < 0.001) compared to diclazuril (Dic) monotherapy (*p* < 0.01), atovaquone (Atov) monotherapy and combined diclazuirl plus atovaquone (Dic + Atov) therapy (*p* < 0.001) and normal sham controls (Co). **(B)** Hepatic pathological score distribution in *Toxoplasma* (Tox) infected dams (*p* < 0.001) compared to diclazuril (Dic) monotherapy (*p* < 0.01), atovaquone (Atov) monotherapy (*p* < 0.05), combination diclazuirl plus atovaquone (Dic + Atov) therapy (*p* < 0.001) and normal sham controls (Co). Pathological slides were stained with H&E. (*n* = 6–8/group).

Hepatic structures of infected-sham treated dams enlarged twofold and increased in weight due to a substantial inflammatory response to the organisms (*p* < 0.001) **Figure 4A**. Pathological investigation demonstrated severe hepatitis with infiltration of inflammatory cells, multinucleated dysplastic hepatocytes, giant cell transformation, stellate cells activation and hepatic cells necrosis (pathological mean score of 3.5 from 4 most severe) **Figure 4B**. Combination therapy with diclazuril plus atovaquone exerted unique synergism and preserved hepatic appearance, weight and microstructure (*p* < 0.001) and to a lesser degree, diclazuril monotherapy (*P* < 0.01) and atovaquone monotherapy (*p* < 0.05) prevented *Toxoplasma* induced hepatitis (**Figures 4A,B**). Overall, these effects of combination therapy present an striking synergy between two structurally distinct compounds in protecting architecture from exaggerated inflammatory reaction.

### *Toxoplasma* INDUCED PANCREATITIS

**FIGURE 5:**
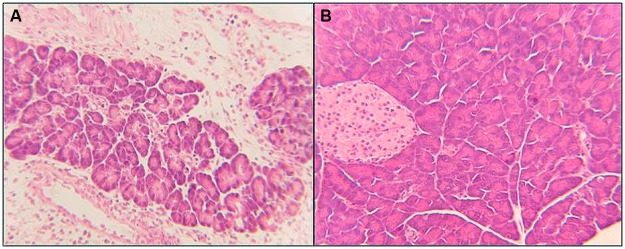
**Pancreatic section from *Toxoplasma* infected and treated dams stained with H&E. (A)** Pancreatitis: demonstrates loss of microstructure, degeneration, and necrosis of pancreatic cells, degeneration and loss of islets, replaced with infiltration of inflammatory cells. **(B)** Combination diclazuirl plus atovaquone (Dic + Atov) therapy protected pancreatic architecture against inflammatory and infectious response, and preserved panc and beta cells, and islet’s microstructure. (*n* = 6–8/group).

This was consistent with moderate to severe *Toxoplasma* induced pancreatitis in infected dams (*p* < 0.05) with infiltration of inflammatory cells, vacuolization, degeneration, and necrosis of pancreatic cells followed by the degeneration and loss of beta cells and islets (**Figure 5A**). Combination therapy with diclazuril plus atovaquone therapy and monotherapy protected dams from these inflammatory and pathological aspects of pancreatitis (**Figure 5B**) and gastrointestinal toxoplasmosis.

### CONGENITAL TOXOPLASMOSIS

Infected dams had nested smaller fetuses (*p* < 0.001) and sporadic preterm labor or stillbirth. Combination therapy diclazuril plus atovaquone as well as monotherapy with atovaquone similarly and to a lesser extent diclazuril monotherapy (*p* < 0.01) protected nested fetuses from retardation and demise (**Table 1**). In addition, uteri considerably augmented owing to accumulation of inflammatory fat, influx of inflammatory cells in infected-sham treated dams and *Toxoplasma* organisms were detected in Giemsa stained and IHC slides (not shown). Combination therapy with diclazuril plus atovaquone improved the infectious inflammatory response and edema but with no significant changes in the uteri weight, presumably due to the increased number of healthy fetuses (not shown).

### TOXOPLASMOSIS AND ABDOMINAL HYPERSENSITIVITY

Finally, pain related abdominal hypersensitivity significantly elevated in *Toxoplasma* infected-sham treated dams manifested with severe abdominal withdrawal and excess grooming in comparison to normal sham control dams (*p* < 0.05). Combination diclazuril plus atovaquone therapy and atovaquone monotherapy preserved the normal abdominal response to von Frey stimuli (**Table 1**). However, diclazuril monotherapy had no significant effect on the dams’ response to the mechanical stimuli.

## DISCUSSION

*Toxoplasma* is a leading cause of foodborne diseases, congenital complications, morbidity and mortality. Yet, toxoplasmosis is an underestimated syndrome and usually detected in autopsy or remains undetected due to the non-specific symptoms and lack of clinical awareness of healthcare individuals (Munir et al., 2000). *Toxoplasma* organisms are transmitted through consumption of undercooked meat, milk and dairy product contaminated with cysts forms. However, the predominant source of *Toxoplasma* infection is considered as vegetables, and fruits contaminated with oocysts from the cat feces in the field (Oz, 2014). In addition, contaminated water is reported as a major source for infection during pregnancy in rural area (Andiappan et al., 2014). Considering high number of cats (>93 million) residing in households in the USA, immunocompromised individuals, and expecting moms, as well as the increasing obese and/or diabetic population are at a high risk of developing toxoplasmosis (Esch and Petersen, 2013). Therefore, awareness of healthcare communities as well as individuals is necessary to contain stray cats, and prevent pets from infection in order to protect the owners from imminent complications.

Toxoplasmosis is a “forgotten disease of vulnerable and poverty” which infects the many in rural (Hotez, 2008) as well as urban area. While, poverty persists, obesity has become a cosmopolitan complication with undetermined pathogenesis. This investigation reports accumulation of excessive infectious and inflammatory adiposity and pathological weight gain in *Toxoplasma* infected dams. *Toxoplasma* association with obesity was supported in a clinical trial with 999 psychiatric healthy normal subjects with exclusion of those with personality and serious mental disorders which have strong association with toxoplasmosis as well as obesity (Reeves et al., 2013). Individuals with positive anti-*Toxoplasma* antibodies had twice the odds to be obese compared to seronegative individuals. Further, obese individuals had significantly higher anti*-Toxoplasma* IgG titers compared to those who were not obese (Reeves et al., 2013). In contrast, no relation with obesity and anti-*Toxoplasma* IgG titers was reported in a trial with confounding factor of excluding individuals over 45 years of age when subjects mostly are prone to develop toxoplasmosis reactivation and obesity (Thjodleifsson et al., 2008). *Toxoplasma* may alter weight gain by reducing muscle lipoprotein lipase and modulating tissue lipoprotein lipase activity during chronic infection to promote triglyceride distribution in adipose tissue (Picard et al., 2002). From 1227 Mexican Americans tested for anti-*Toxoplasma,* 110 (9%) were found seropositive. In fact, this population commonly suffers from high rates of chronic inflammatory diseases, obesity and type-2 diabetes, further suggesting a correlation between toxoplasmosis and these chronic complications (Rubicz et al., 2011).

Toxoplasmosis may manifest with clinical symptoms of acute or recurrent abdominal pain and pancreatitis (Parenti et al., 1996). Chronic progressive pancreatitis may be associated with fat necrosis, obstruction of bile duct, focal hepatic necrosis, elevated amylase and lipase serum values, and abdominal fat. Similarly, in this study infected dams developed increased abdominal inflammatory pain related modifications and severe pancreatitis and hepatitis. There is an association of *Toxoplasma* infection with liver cirrhosis. While, severity of toxoplasmosis complications depend on the immune status of the patient and the strain. Acute *Toxoplasma* infection in mice with RH strain reveal a significant correlation between the increased number of hepatic stellate cells and the amount of *Toxoplasma* antigens, representing an active role for hepatic stellate cells in the pathogenesis of *Toxoplasma-*induced hepatitis (Atmaca et al., 2013). Moreover, the prevalence of anti-*Toxoplasma* IgG is significantly higher among the primary biliary cirrhosis patients (71%) compared with controls without cirrhosis (40%, *p* < 0.0001), whereas the infection burden is rare in healthy subjects (20% vs. 3%, respectively, *p* < 0.0001). It is predicted that *Toxoplasma* to increase the risk of primary biliary cirrhosis in patients (Shapira et al., 2012). Since, latent infection is fairly common, and once infected organisms reside for the lifelong; the *Toxoplasma* interventions with safe and effective regimens will have a great impact on health related concerns in vulnerable individuals.

Available treatments for toxoplasmosis, sulfasalazine, pyrime thamine, sulfadiazine, and spiramycin, have major side effects and not always effective. Seroconvert pregnant women are treated with spiramycin to reduce the risk of fetal placental transmission. However, spiramycin treated patients retain *Toxoplasma* DNA in peripheral blood and remain infected (Habib, 2008). In addition, spiramycin is effective only in early pregnancy and not after organisms penetrate the placenta and fetus (Julliac et al., 2010). In a 20 year prospective trial of infected moms treated with spiramycin alone or combined with pyrimethamine-sulfadoxine, 17% of newborns had established congenital toxoplasmosis and 26% developed chorioretinitis after birth (Berrebi et al., 2010). In another study the transmission rates of toxoplasmosis were 7% in the first, 24% second, and 59% in third trimesters, respectively, for infected mothers treated with combination spiramycin and pyrimethamine-sulfadoxine (Bessieres et al., 2009).

Because of these shortfalls, there is urgent need for more effective therapeutic modalities with no toxicity to encounter congenital as well as recurrent toxoplasmosis. In this investigation combination of diclazuril plus atovaquone therapy synergistically protected dams and fetuses from severe complications of toxoplasmosis including gastrointestinal and the inflammatory adiposity accumulation.

Atovaquone (hydroxy-1,4-naphthoquinone) an standard of therapy against acute toxoplasmosis is not approved for congenital infection. Atovaquone suppresses mild gastrointestinal toxoplasmosis in pregnancy model (Oz and Tobin, 2012; Oz, 2014). However, atovaquone monotherapy is not effective against severe complications of colitis, hepatitis and splenits and inflammatory fatty deposits as shown here.

Additionally, diclazuril (4-chlorophenyl [2,6-dichloro-4-(4,5-dihydro-3H-3,5-dioxo-1,2,4-triazin-2-yl)pheny l] acetonitrile) is used in livestock to prevent coccidiosis and equine infection with *S. neurona*. Diclazuril is orally absorbed with steady-state concentrations in plasma and cerebrospinal fluid (CSF) to inhibit the proliferation of 95% of the organisms (Assis et al., 2010; Oz, 2014; Oz and Tobin, 2014). Dicalzuril exclusively binds and affects *Toxoplasma* organelle for photosynthetic reaction center (protochlorophyllide) containing a trace of chlorophyll (Hackstein et al., 1995). This herbicidal-binding site for diclazuril is highly specific for *Toxoplasma* and other Apicomplexans, providing an exceptional chemotherapeutic sensitivity. Therefore, diclazuril binds the chloroplast epitopes and interacts with the D1 protein, with no intervention with the mammalian cells. In addition, diclazuril downregulates expression of serine/threonine protein phosphatase and causes apoptosis of *Eimeria tenella* merozoites (Zhou et al., 2013). Serine/threonine protein phosphatase (EtRACK) has 98% homology with *Toxoplasma* with a predicted mechanism of action for diclazuril efficacy against toxoplasmosis. Diclazuril dose dependently protects against moderate feto-maternal gastrointestinal complications in model (Oz, 2014; Oz and Tobin, 2014). Yet, combination diclazuril plus atovaquone therapy has superior synergic effects against toxoplasmosis in comparison to diclazuril and atovaquone monotherapy. As such, combination therapy with a promising safety and efficacy proven in the most vulnerable group during “fetal maternal” stages can be applicable as a preventive measure in the endemic areas specifically in pregnancy as well as in pets. It is anticipated that the novel combination diclazuril plus atovaquone therapy to be as effective in maternal congenital as well as acute and chronic persistence CNS and ocular toxoplasmosis in patients.

## CONCLUSION

Diclazuril plus atovaquone combination was safe with a novel therapeutic synergism protected dams from inflammatory and infectious colitis, pancreatitis, obesity and other pathological complications as well as preserved the fetuses against congenital toxoplasmosis. The future trials will prove the anti-toxoplasmonsis properties of diclazuril plus atovaquone combination in acute or chronic ocular, CNS and congenital toxoplasmosis in patients.

## Conflict of Interest Statement

The author declares that the research was conducted in the absence of any commercial or financial relationships that could be construed as a potential conflict of interest.

## Abbreviations

BSA: bovine serum albumin
CDC: center for disease control
CNS: central nervous system
EPM: equine protozoal myeloencephalitis
FDA: Food and Drug Administration
H&E: hematoxylin eosin
IACUC: institutional animal care use committee
IBC: institutional biosaftey committee
IHC: immunohistochemical staining
i.p: intraperitoneally
MEM: minimum essential medium
NIH: national institute of health
PBS: phosphate buffer saline
*S. neurona*: *Sarcocystis neurona*
Tox: *Toxoplasma gondii*.
